# The ‘Spanish’ influenza pandemic: new evidence for influenza outbreaks in England and France prior to 1918

**DOI:** 10.1017/mdh.2025.10053

**Published:** 2026-07

**Authors:** Douglas Gill, John Oxford

**Affiliations:** 1 Independent Scholar; 2 Blizard Institute of Cell and Molecular Science, UK

**Keywords:** Influenza, Pandemic, Origin, Spillover, Mutation, Cyanosis

## Abstract

The Spanish influenza pandemic of 1918 caused well over fifty million deaths. The epicentre undoubtedly was China, where gene mixing of different virus strains occurred amongst aquatic, migrant birds. But where and when did the virus first infect (or spill over to) a human being? We take, as our starting point, a paper demonstrating that an infection causing the same symptoms as the influenza virus was widespread in New York during the winter of 1917–1918. The authors of that paper went on to suggest that the virus had probably reached North America from Europe, in the context of troop movement during World War I. Our own researches have focussed on this point. We show that outbreaks of serious respiratory disease, local in nature but causing unusual patterns of mortality, were indeed reported by scientists and doctors in army hospitals in England and in France, well before the first wave of the pandemic had arrived. We use the records of these hospitals, now held in the National Archives, to trace the progress of this disease amongst the individuals who fell ill. We examine contemporary reactions to this minor epidemic – an epidemic, we suggest, which acted as a herald wave of the pandemic yet to come. The latter part of our paper addresses the second question, as to how troop movement across the North Atlantic, once the United States had entered into war, may well have enabled the virus to spread from Europe to North America.

## Introduction

At some point, more than a century ago, the virus H1N1 transferred, or ‘spilled over’, from a bird to a human being. Shortly thereafter, the world was engulfed in a pandemic. This pandemic, of so-called ‘Spanish’ influenza, represents the most deadly wave of death and disease that mankind has known. Yet, the question remains as to where and when this spillover event most likely occurred. The genetic mixing bowl, and thus the ultimate origin, of influenza viruses is usually to be found amongst the migratory geese and ducks of western China. In this instance, the debate relates not to the origin or source of the virus, but to the place and date of its transfer. Did the spillover event take place in China itself, in the American Midwest, or in Western Europe?

The suggestion that the spillover event occurred in China rests principally upon the reports filed by Chinese officials working at the time.^1^ These officials, based in the Treaty Ports, played medical and administrative roles, but, being neither scientists nor working physicians, they supply an incomplete picture of the clinical symptoms involved. And their reports shed but a glimmer of light on the health of those vast populations who lived further inland. Indeed, when Chinese labourers, recruited inland, started disembarking in France, they appeared to have had no prior exposure to pandemic influenza.^2^

A second hypothesis, and one that used to be widely accepted, is that the pandemic emerged in North America in the spring of 1918. This hypothesis, first proposed by the historian Alfred Crosby, was then amplified by John Barry, a writer and historian.^3^ Barry describes how a family doctor reported on a handful of cases of influenza, probably of the seasonal type, amongst scattered farmsteads in Kansas. But Crosby himself, the author of a respected history of the pandemic, insisted that, in order to confirm the first outbreak of a new pandemic virus, it would be necessary to have the authority of a scientific paper written by contemporary pathologists and bacteriologists. The outbreak in Kansas falls short of this mark. There were no qualified scientists on hand at the time, and no data survives on incidence and mortality. A lack of data apart, the Kansas hypothesis has been fatally undermined by the work of a team of epidemiologists led by Daniel Olson. Olson describes an outbreak of influenza in the city of New York during the winter of 1917–1918.^4^ He emphasises the relative youth of the victims and the unique clinical symptoms. This outbreak predates the Kansas report. Olson concludes that the Kansas hypothesis has for long been accepted ‘without rigorous reevaluation of the original evidence’.^5^ His findings, he suggests, ‘reopen the possibility that the virus had spread from Europe to New York City in the context of troop movement during World War I’.^6^

Echoing Olson, we looked more deeply into the central question which his paper has raised. Could outbreaks of serious respiratory disease, local in nature but causing unusual patterns of mortality, have indeed been reported by scientists and doctors in Europe before the influenza pandemic arrived? And, if such reports had been published, might their language have betrayed a certain puzzlement, in the minds of the authors, as to the aetiology of the disease which they had seen? Acting on this imperative, we conducted a series of searches. We read through, page upon page, the leading medical journals in England, France, Germany, and the United States.^7^ As a result, we identified papers whose findings suggested that two such outbreaks had in fact taken place. The first was published by British Army physicians, serving in northern France, in mid-1917,^8^ and the authors of the second, also army physicians, reported on an identical problem occurring at much the same time, in hospitals in the south of England.^9^

The language employed in the second such paper is perhaps the more worthy of note. Its authors were amongst the most illustrious medical men of the day. Adolphe Abrahams, the founder of sports medicine in the UK, headed the list, whilst Herbert French ably assisted – the same French whose clinical work on ‘differential diagnosis’ is in its sixteenth edition today.^10^ They had encountered cases, they wrote, of a ‘definite type’: a type which, while ‘clinically recognisable almost at sight’, had yet differed ‘from anything we have been familiar with in men of military age in civil life’.^11^ And, as if to justify the need for publication, they sought to draw attention to these cases for ‘two chief reasons’. ‘In the first place, the affection has a very high mortality – very much higher than that of lobar pneumonia; secondly, there are features and circumstances which suggest an epidemic nature’.^12^ On two of the symptoms, they laid particular note. They wrote, first, of the dyspnoea (shortness of breath), as the cause of ‘great respiratory distress’; and, second, of ‘the peculiar heliotrope cyanosis of the face’.^13^ It was this facial hue, of course, which, some eighteen months later, was so widely seen amongst those influenza cases which ended in death. It is viewed, along with the relative youth of the victims, as defining the clinical description of the Spanish influenza.

## Could advance warning have been given of an approaching pandemic?

In 1917, Dr Abrahams and his colleagues were engaged in the care of the thousands of the wounded and sick flowing back from the Front, and, accordingly, had not had the chance to take a meticulous count of the numbers involved. They emphasised, though, the ‘epidemic nature’ of the disease, and the exceedingly high rate of mortality – ‘something like 50 per cent’ – in the ‘scores’ of cases which they had seen. They were puzzled as to the condition with which they were faced. They could not be certain. Even so, ‘the conjecture is that the disease starts as an influenzal infection, terminating in the fatal cases as pneumococcal septicaemia’.^14^

Drs Abrahams and French acknowledged that they were not the first researchers to publish in this field. That honour goes to two physicians and a pathologist, comparatively young men, whose paper on the same subject was published in July 1917. They were John Hammond, William Rolland, and Thomas Shore.^15^ Still, behind the pioneering nature of the work they undertook, there lay the guiding influence of an older, more experienced hand. He had not only prompted their research but had brought their team together in order to achieve it. In a postscript to their paper, the three authors acknowledge their debt to ‘Sir John Rose Bradford, K.C.M.G., who originally pointed out to us the distinctive nature of these cases’. Bradford had served in France since the beginning of the war. As a consulting physician to the British Expeditionary Force, his duties included the inspection of army hospitals across the Western Front.^16^ There were scores of such hospitals, and at Etaples, for instance, where Lieutenant Hammond and Captain Rolland, of the Royal Army Medical Corps, were working in 1917, a dozen or so had been gathered, whose accommodation extended to some twenty thousand beds.^17^ Bradford’s role was central. Perhaps, as the paper maintained, Hammond and Rolland had been struck by patients coming into their wards in December 1916, suffering from an ‘unusually fatal disease’, perhaps they had noticed that these men did present ‘a symptom complex so distinctive as to constitute a definite clinical entity’ (such as influenza)^18^: but, as a mere lieutenant and captain, surrounded by scores of majors and colonels, they would have been powerless to do more by way of research had they not had assistance of a most powerful kind.

Bradford, the paper’s postscript continues, gave us ‘much kind assistance throughout the investigation’.^19^ Bradford, in fact, did rather more than assist. The team carried out ‘156 consecutive necropsies during February and early March, 1917’^20^: an autopsy, that is, of every soldier who died at Etaples of a medical issue during this period. However, prior to the programme’s inception, Hammond and Rolland appear to have lacked the requisite skills. Hammond was an internal medicine specialist and Rolland a histopathologist; and so, in an unusual move, Shore, a morbid anatomist, was brought to Etaples just days before the work was scheduled to begin. Our research in the War Office archives makes the picture clear. Shore reached No. 24 General Hospital, Etaples, on 27 January 1917.^21^ By then, the groundwork for the programme of necessity was done; the required laboratory and mortuary facilities arranged and laid out; and so, with Lieutenant Shore’s arrival, the final cog was thus slid into place. Bradford alone could have pulled the levers required to bring this about. One source has described the interplay between the two men. At Etaples, it explains,

‘Dr. T.H.G. Shore …was in charge of the post-mortem room and laboratory for the whole base, and many thousand post-mortem examinations were made there. Sir John Rose Bradford … visited the rooms every day and discussed the findings with Shore and anyone else present’.^22^

We suggest that this account is inflated. Shore was simply appointed ‘Officer in Charge, Mortuary, Etaples Administrative Area’, and there were more than a dozen laboratories within very close reach.^23^ And again, as a consultant physician, with scores of hospitals to inspect and advise, Bradford could not have made everyday trips to Etaples. Even so, it points to the role which Bradford undoubtedly played. He created a new post at Etaples and appointed Shore as its holder. And he gave him, as his first major task, the job of working with Hammond and Rolland.

Hammond’s paper describes an exhaustive piece of research. During February and March, 1917, he and his colleagues conducted ‘156 consecutive necropsies’. They discovered thereby the ‘prevalence of bronchitis of a purulent type’. It occurred ‘as a primary condition in 45 and as a secondary condition in 26 of the cases examined. Altogether purulent bronchitis was found in 45.5 per cent of the total cases’.^24^ And they listed, as the first of the ‘typical’ appearances of those who had died, the fact that ‘The face is more often than not cyanosed …’^25^ Again, unlike the lingering decline which might have confronted a fit and active young man who, untypically, succumbed during a case of bronchitis, the end, in the case of these purulent patients, was often quite swift. ‘The patient usually dies … on the fifth or sixth day’.^26^

‘Bronchitis’, of course, is a clinical term, and describes a condition which, at its root, can have a number of causes. So, beyond conducting autopsies and examining the organs of those who had died, Hammond *et al.* carried out a series of laboratory tests on the sputum of the men who fell ill. In particular, twenty specimens of sputum were subjected to detailed bacteriological examination. The authors were careful to note that these twenty were taken ‘from cases which in their clinical aspect differed from cases of ordinary bronchitis, and most of which presented many of the signs and symptoms [cyanosis, etc.] described in another part of this paper’.^27^ In seventeen of those cases, a bacterium then known as *Bacterium influenzae* was detected – a bacterium now known not to cause influenza, but to play a role as a secondary invader.^28^ In thirteen cases, pneumococcus – a common cause of pneumonia – was found. In addition, in a small number of cases, other pathogens were detected as well. In conclusion, the authors opined, ‘we consider’ the cause of the disease to be *B. influenzae* ^29^; but, more important than that, from the perspective of scientific knowledge today, they pointed to the ‘well-marked clinical features which distinguish these cases from ordinary cases of bronchitis’. The most prominent they listed as ‘the characteristic sputum, the extreme tachycardia, the cyanosis, the course of the temperature (notably the ante-mortem fall), and the extremely high mortality’.^30^ Overall, Hammond concluded, the disease had ‘assumed such proportions as to constitute almost a small epidemic’.^31^

In their Aldershot hospitals, in southern England, Dr Abrahams and his colleagues were unaware, initially, of Hammond’s work at Etaples, but, early that summer, they felt impelled to describe the peculiarities of the condition which they had seen. They, too, employed the words ‘purulent bronchitis’^32^ and were chiefly struck by the ‘very high mortality – very much higher than that of lobar pneumonia’, and by the idea that the condition was epidemic in nature.^33^ They published the clinical notes, and the bacteriology, in regard to eight cases, all of which had arisen during the months of March, April, and May 1917. They admitted, when their paper came to be published, that theirs was a less ‘thorough and elaborate publication’ than that which had flowed from the work at Etaples. ‘We have, however, published our own results, if only to confirm the findings of Hammond, Rolland, and Shore’.^34^

## Why did the new infection not spread more widely?

One hundred years later, research into these findings can still be pursued. Abrahams appends, as an afterthought to his paper, the view that, quite probably, ‘the disease in question is more widespread than has hitherto been recognised’.^35^ And Olson, too, is intrigued by the notion of ‘spread’: ‘the possibility that the virus had spread from Europe to New York City in the context of troop movement during World War I’.^36^ And thus the *leitmotiv* which has informed our own work in this field. Did the condition first noticed in December 1916 by Hammond and Bradford spread to other locations in France, and is there evidence that, in turn, it spread via troop movements to New York and elsewhere?

We chose, in the first instance, not to answer these questions head-on. ‘Troop movement’ is a very broad phrase, and when, in a single month in 1917, some tens of thousands might cross the Atlantic (albeit from North America to France),^37^ or some 68,904 soldiers be ‘despatched’ from Etaples to serve at the Front,^38^ the logging looked daunting indeed. Instead, we have followed a narrower path. It has proved possible, via the War Office archives, to re-examine, one hundred years later, the findings upon which Hammond’s paper was based; and, in particular, to unearth some of the notes which he and his colleagues composed. These three physicians conducted ‘156 consecutive necropsies’, and we have sought to re-interpret, in the light of what science can tell us today, the conclusions to which they were drawn. And, just as important, from the viewpoint of ‘spread’, we have sought to retrace some of the journeys undertaken by these 156 soldiers as they were brought into the hospital by an ambulance vehicle or train. The question remains: did the disease establish itself in the trenches, or was it the result of a spillover from pigs or birds within the encampment itself?

As it happens, we were acquainted, through previous projects, with the records of the hospitals which had served at Etaples.^39^ And we had interrogated, in the closing years of his life, Major O.C. Guinness, the Adjutant at Etaples, who was responsible, with the commandant, for the administration of the base.^40^ We knew, from the War Office archives, that both the hospital registrars and Major Guinness’s office had kept their records in a meticulous state. It came as no surprise, therefore, to find, in those self-same archives, the clinical notes and the post-mortem findings in regard to many of those who had been examined in Dr Shore’s ‘mortuary tent’. And there was further information to hand. Today’s medical historian has one important advantage, as compared with the situation confronted by Dr Hammond *et al*. In 1917, an army pathologist could research the bacteriology and examine the organs, but he lacked the full medical and service record of the patient him- or herself. These documents did not follow the soldier, but were kept safe at the office for Infantry Records in the UK. Such records could be quite comprehensive. They might detail the state of his health when the soldier enlisted, give a history of illness and treatment, specify the locations at which he had served, and even log journeys by ambulance train.

In the year 1917, the subject of data protection was not the sensitive matter which it later became. While Dr Hammond was conducting ‘a study of 156 consecutive necropsies made during February and early March, 1917’, Major Guinness and his staff were recording careful details in regard to those who had died. A list of those patients whom Hammond sent for *post-mortem* examination, therefore, has proved a simple one to construct. One hundred and fifty-six men and one woman died at Etaples, from causes other than wounds, during the period beginning on 1 February and ending on 19 March 1917.^41^ Four men died of ‘sickness’ on the first day of February – Sapper Alfred Elmore, Privates Henry Hague and John Hill, and Lance Corporal George Godwin – and it seems highly likely that these patients comprised the first of the series described by Hammond *et al*. Again, Hammond, having noted that ‘20 specimens of sputum have been submitted to the laboratory for bacteriological examination’, then permitted *The Lancet* to publish some notes from the labels attached to each one.^42^ Identification, in some of these cases, has followed with ease.

## How the first cases of this new disease were identified

Amongst the specimens sent to the laboratory, there is one which bears the name ‘Pte. U.’. This must have been taken from No. 14075 Private Harry Underdown, 12th Battalion, West Surrey Regiment.^43^ He died in No. 24 General Hospital, Etaples, on 21 February 1917. In Underdown’s case, Pfeiffer’s bacillus was present to a minor degree, but Pneumococcus, *D. catarrhalis*, and a Gram-positive diplococcus were detected as well. Overall, ‘widespread broncho-pneumonia’ characterised the state of Underdown’s lungs.^44^ If Hammond *et al.* had sought more information on Underdown’s past, they had only one source, and that was what the soldier himself might recall. His medical records remained in England throughout. And, given his past, Underdown’s own recollections may well have been blurred. Today, we can give the full story of what had occurred: a story of considerable interest, in that Underdown can be regarded as amongst the first cohort of victims of a new and emerging pandemic. A fit man on enlistment, this soldier had made his first crossing to France in August 1916.^45^ In October, however, he had been buried alive by a shell.^46^ His condition was serious, sufficiently so, in fact, that he had been brought back to England for treatment. On admission to hospital – a converted workhouse, it seems^47^ – he was found to be ‘very shaken’ and suffering from ‘loss of speech and memory’. His condition was described as ‘nervous prostration’.^48^ After three weeks of ‘rest and bromides’, he returned to duty, and then, in February 1917, he was sent back to France.^49^ Nothing more is known about the last days of Underdown’s life, save that on 21 February 1917, he died at Etaples, and that the death certificate gave the cause as ‘bronchitis’.^50^ In *The Lancet*, of course, Hammond more carefully described what he and his colleagues had seen. In its clinical aspect, Underdown’s case ‘differed from cases of ordinary bronchitis’ and ‘presented many of the signs and symptoms’ associated with the purulent kind.^51^

A second case, from the series which Hammond constructed, should be scrutinised at this stage. It concerns a man who fell ill at much the same time, whose illness threw up a profusion of paper, and whose cause of death prompted queries in distant Whitehall. In early January 1917, No. 23/455 Private Walter Scott was to be found serving with an infantry unit in or near the front line. ‘A well-nourished, well-developed male about 33 years of age’,^52^ he reported sick.^53^ The problem was sufficiently serious for him to be taken out of the line. He was sent to a ‘casualty clearing station’ – a field hospital, one would call it today. Four days later, Scott was ‘not improving’, and he was put on an ambulance train, destined for Etaples.^54^ Slowly, the train moved across France. It reached Etaples on 16 January, where he (and four hundred others) were conveyed to vacant hospital beds. That day, Captain J.C. Tull, of the Canadian Army Medical Corps, opened a ‘medical case sheet’ and, at its head, entered Scott’s name. Tull spelt out the patient’s condition as day followed day.

‘Onset of illness about 6 days ago, with chilly sensation, headache and general pains, dry cough, slight sore throat. Headache … persists – patient feels weak… Throat hyperaemia. Lungs and heart clear’.^55^

Two weeks later, the patient seemed close to being discharged. ‘2/Feb/17. Temp normal since Jan 30th. Slight dry cough. Weak – otherwise comfortable’.^56^ However, Scott’s recovery stalled. The patient was now transferred from tented accommodation to a bed in a hutted ward: perhaps because of the extra warmth, and the more careful nursing, which a hut could afford. But to no avail.

‘14/Feb/1917. Severe cyanosis. Whole of right lung shows signs of consolidation. Sharp crackles all through left lung ….

15/Feb/17. Increasing dyspnoea throughout last evening. Pale, cyanosis. Oedema of lungs. Death 1.15 am’.^57^

What had caused a patient who having had a mild respiratory illness and had progressed to the recovery stage thereafter to undergo a relapse? And then, within a matter of days, to be dead? Overall, Scott’s death certificate supplied only two words: ‘Lobar pneumonia’. But, on the Medical Case Sheet, Tull sought to probe deeper: ‘Influenza. Complications – Lobar Pneumonia, Nephritis, &c.’.

Still, whatever the details, the circumstances surrounding Scott’s death did not find favour in War Office eyes.^58^ No. 1 Canadian General Hospital was ordered to ‘furnish a medical report on the case’.^59^ The exchange was unusual. Every day, throughout the Great War, reports on hundreds, sometimes thousands, of deaths were reaching Whitehall, and it was exceedingly rare for the question of cause to be raised.^60^ What, then, had caused the request for further and better particulars, in regard to Scott’s death? The Archives are silent on this. Of course, eighteen months later, when the pandemic arrived, a man or woman who had contracted influenza, who seemed to recover, but in whose lungs consolidation had then taken place, would have been but one case in perhaps tens of millions, and the cyanosed facies was simply a symptom which, by late 1918, every mortician was seeing again and again. In February 1917, though, this condition was the cause for remark. Even so, the question remains as to why neither Sir John Bradford nor his War Office colleagues seemed to suspect that the deaths which they had observed at Etaples, and whose significance they clearly discerned, might be the first wave of an approaching influenza pandemic. Or, if they did so suspect, why did they not instruct Hammond or Tull to explore this line of enquiry?

A number of answers to these questions suggest themselves, we submit. In the first place, nineteenth-century physicians were not necessarily taught to regard influenza as a separate disease. In England, for instance, an influential volume, *Annals of Influenza*, had been published on the subject by Theophilus Thompson. In its pages were to be found accounts of every influenza pandemic reported by medical science since the year 1510. In 1890, as the Russian pandemic drew to a close, the book was republished, but its title was changed. It was now entitled *Influenza or Epidemic Catarrhal Fever*,^61^ a reflection of the uncertainty prevailing in its author’s mind as to the underlying nature of influenza itself. Of course, Bradford’s generation of physicians had grown up under the shadow of ‘important discoveries with the microscope … recently made by German investigators’.^62^ One such discovery was made by Richard Pfeiffer.^63^ His claim, in 1892, that he had identified *B. Influenzae* as the pathogen which caused influenza was immediately assailed; and though twenty-five years later, Pfeiffer’s views had their supporters, many scientists remained unconvinced.^64^ There was scepticism, too, in regard to influenza’s importance, and, in particular, as to whether it constituted a separate and distinct disease. Thus, a textbook on the diseases of the lungs, published in Leipzig in 1916, contained no substantial entry under ‘influenza’, although its 360 pages described every other condition.^65^ Equally, the pages of the fortnightly *Bulletin de l’Institut Pasteur*, published in Paris, contained, in the whole of the year 1917, only one modest reference to ‘grippe’. And then, on the ground, in May 1918, as the first wave of the pandemic began to take hold, a senior physician, commanding a large army hospital in France, could still write of ‘so-called “influenza”’.^66^

A second reason may well have discouraged Bradford and Abrahams from seeing influenza as the disease which underlay the problems they faced. The disease now appearing at Aldershot and Etaples did not show the one characteristic which influenza was usually thought to have had: high spreadability in the community. Theirs was a misapprehension, a misapprehension which, strongly rooted a century ago, still finds adherents today. A recent account of the Spanish influenza pandemic suggests that these outbreaks in the year 1917, as reported on by Hammond *et al.*, could not have been influenzal in nature.

‘There are problems with the Étaples theory, though: there are no records of outbreaks in the civilian population of northern France at that time. It seems odd that a dangerous infectious disease would erupt simultaneously at a number of military bases, whilst the civilian communities between them remained unaffected – especially since we know that the camp at Étaples lived in “osmosis” with the town’.^67^

But the ‘oddness’ is more apparent than real. Molecular pathology suggests that a potentially emerging bird influenza virus replicates in the lower respiratory tract of patients; it is not completely adapted to humans. Until it can replicate in the upper respiratory tract, following mutations in the virus genome, person-to-person transmission will but rarely occur.^68^ A recent example will make this quite clear. Thus, during the potential emergence and spillover of bird influenza A(H5N1) in Asia in 2003, it was found that the case fatality in patients could exceed forty per cent without any sign of enhanced spreadability in the community at large.^69^ In that outbreak, which took place in Hong Kong, the virus did not progress to the next stage and adapt itself to humans. So the world was spared an influenza pandemic.

## The role of the official Censor

We suggest that a third reason may have underlain Bradford’s reluctance to weigh the influenzal basis of the disease. The views of the Censor would have been much on his mind. Without the Censor’s approval, Hammond’s paper would have had to be shelved. Indeed, once hostilities finished, and it was time to look back, physicians saw cause to regret the paucity of written material upon which they could work. ‘Papers published during the war were comparatively few’, the British official history lamented, and this scarcity its editor attributed to ‘the general military policy which of necessity governed the publication even of medical reports’.^70^ In no way, it was clear, would ‘general military policy’ have permitted the publication of papers which might lead the reader to think that the British Army faced problems which it was unable to solve. And, although Hammond’s researches did find their way into print, the results of the Censor’s excisions remain clear on the page. In the title of his paper, the hospital name is redacted, and no location is given, save that it was somewhere ‘in France’.^71^ Questions of influenza apart, until 1918, the Censor never permitted the word ‘Etaples’ to appear in the press. After all, there were gathered, around that small town, the largest ammunition depôt, the largest reinforcement camp, and the largest hospital complex, which the British Army maintained throughout the Great War.

The hand of the Censor may well have constrained Bradford and Hammond in other ways too. Bradford’s duties involved him in visiting hospitals throughout northern France. We think it unlikely that, during these tours of inspection, he did not make enquiries as to whether respiratory cases of a similar nature were or were not to be seen in their beds. Hammond’s paper is silent on this: unsurprising because, if he had published some comparative figures, he would have had to disclose how his comparisons worked. He would have had to compare the numbers of deaths at Etaples with those occurring at other hospital bases. On balance, therefore, we believe that Bradford and Hammond chose to keep silent, whether they would have wished it or not.

In pursuit of these thoughts, about what Hammond might have been able to write, had he (and Bradford) not been constrained by the Censor, we resolved to make enquiries along similar lines. Accordingly, we set out to revisit, via the War Office archives, the patient records created both at Etaples and at further hospital centres maintained by the British Army in France. We donned Bradford’s hat, so to speak, and entered, during the winter of 1916–1917, the hospitals which he may well have visited himself.^72^ The task was laborious, though fairly straightforward. The first point of call, for those who fell ill or were wounded at points along the Front, was at the nearest casualty clearing station; but such patients who seemed likely to survive were then conveyed to hospital centres located close to the ports. The centre at Etaples was certainly the largest, but, during the winter of 1916–1917, those at Rouen and Boulogne were of a similar size. Each could accommodate, in times of ‘crisis’, more than twenty thousand beds. We chose to go to Rouen. The town lies one hundred miles distant from Etaples, and therefore had purulent bronchitis been occurring widely, the army hospitals here would have thrown up a sufficient number of cases to demonstrate the scale of the problem. In the event, our examination of the records of the Rouen hospitals has provided some striking results, results which could not have failed to alert Sir John Bradford had he too, as we strongly surmise, made this journey himself.

A list of patients dying of disease, in the British Army’s hospitals at Rouen, proved fairly simple to construct. Having constructed this list and examined the cause of death in each case, our findings are clear. During the period stretching from the first day of October 1916 to the last day of May 1917, a total of 226 Rouen soldier-patients, whose surnames fell within the range of A to H, died of causes which, in the main, did not result from injuries or wounds.^73^ The cause of death, in some 154 of these cases, is definitely known, and, of these 154 cases, some 63 may be classed as having died through respiratory disease. This disease, had it been examined at Etaples, might reasonably have been labelled ‘purulent bronchitis’. Sixty-three deaths out of a total of 154: the proportion warrants some consideration. Consider this passage written by Hammond *et al.* at Etaples:

‘The prevalence of bronchitis of a purulent type is seen from a study of 156 consecutive necropsies made during February and early March, 1917, in which it occurs as a primary condition in 45 and as a secondary condition in 26 of the cases examined. Altogether purulent bronchitis was found in 45.5 per cent. of the total cases’.^74^

At Etaples, then, a selection of 156 deaths made undeniably at random – by chronological order of decease – led to the discovery that 45% of all the deaths were associated with this new form of bronchitis. At Rouen, out of 154 cases, selected simply by the accident of which patients’ records survive, some 63 had died, arguably through the workings of this same disease. A proportion of some 41%.

When these sixty-three Rouen ‘respiratory’ deaths are set out on a chart, to show the numbers dying each week, a striking picture is obtained. Two peaks are to be seen. The first during Week No.14 (that is, the week beginning 31 December 1916), and the second during Week No. 21 (beginning on 18 February 1917). The death rate starts at zero in October and falls away to zero in May 1917. The same holds true of seasonal influenza, as seen in the northern hemisphere during the winter months of each year. But, between the peaks in the graph of mortality at Rouen, the death rate falls to nil: a sure sign that there passed through the Rouen hospitals an epidemic wave.^75^

At Etaples, no attempt was made to log the daily level of mortality whilst purulent bronchitis was raging in the camp. Its physicians, when writing to *The Lancet*, give only an impression of its actual rise and fall. Today, by employing the same approach as was adopted at Rouen, many of the deaths can be investigated and a tabulation made. The outcome for Etaples is this:

- During the period stretching from the first day of October 1916 to the last day of May 1917, a total of 214 soldier-patients, whose surnames fell within the range of A to H, died of causes which, in the main, did not result from injuries or wounds.
- The cause of death, in some ninety-four of these cases, is definitely known; and, out of these ninety-four cases, some fifty – more than half – may be classed as having died from purulent bronchitis.

When these fifty cases are set out on a chart, to show the numbers dying week by week, a pattern similar to that occurring at Rouen is seen.

**Chart 1.**
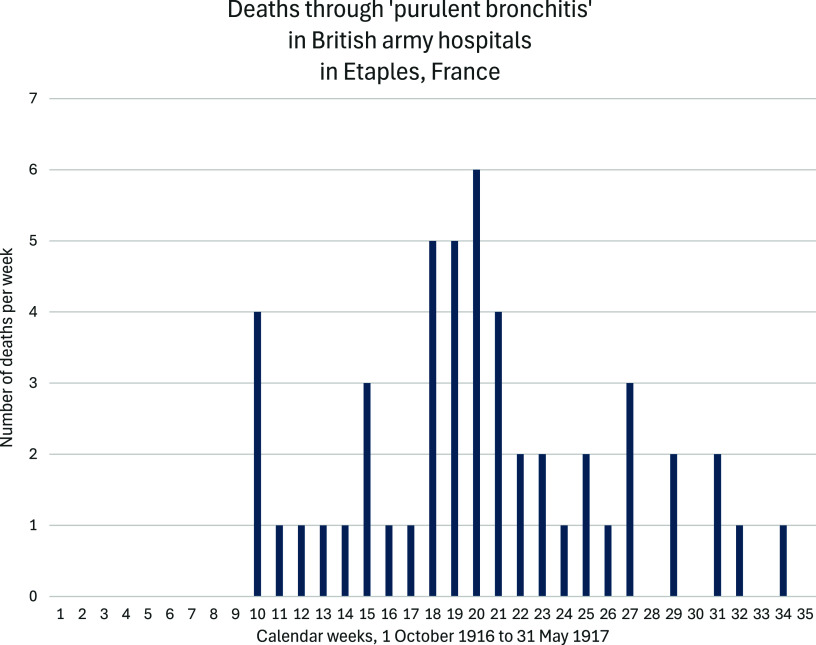
Chart 1. long description.

**Chart 2.**
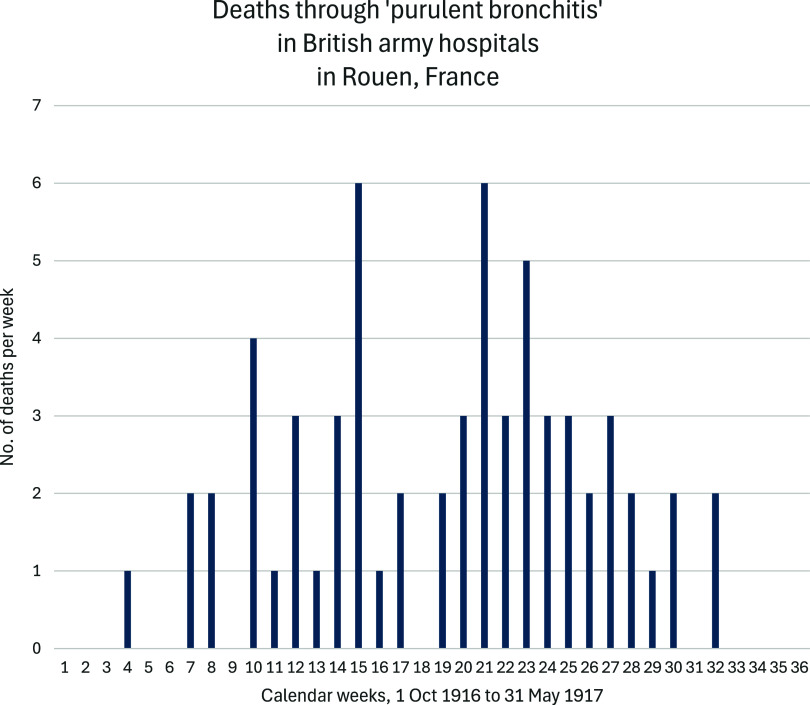
Chart 2. long description.

The inference to be drawn from these two charts is plain (Charts 1 and 2). A respiratory ailment named, at the time, ‘purulent bronchitis’ passed through the Etaples hospitals during the period which ran from December 1916 to May 1917. A chart of its fatal implications demonstrates the existence of an epidemic wave. The same phenomenon, in a form rather more pronounced, passed through the Rouen hospitals during the same winter of the war. At Rouen, it began a few weeks earlier and petered out at some time during April 1917. The lack of spread, though, as compared with what took place next year, during the main wave of the pandemic, is particularly striking. It is illustrated by the disparity between the number of deaths, not exceeding one per day, and the thousands of vulnerable patients gathered in both Etaples and Rouen. There are no data available, however, on mild or sub-clinical infections.

## The identification of an early influenza outbreak in New York

This paper began by acknowledging its debt to the work of Daniel Olson and colleagues. The virus which caused the influenza pandemic of 1918, he writes, could scarcely have originated on the plains of Kansas in the spring of that year. The ‘Kansas’ thesis has to be discarded, he thinks, because the very symptoms which were reported from the American Midwest had already been seen in New York. But Olson then goes one step further. He thinks it unlikely that the virus first appeared on the East Coast of the United States. The failure of the Kansas hypothesis, he writes, taken together with his findings in respect of New York, ‘reopen the possibility that the virus had spread from Europe to New York City in the context of troop movement during World War I’.^76^

We think that Olson’s choice of words raises questions to which no easy answer has emerged. After all, to what ‘troop movement’ can Olson possibly refer? From a North American perspective, the war was being fought in Belgium and France, and, once the United States had decided to take part, there was a need for Washington to enlarge its modest forces, to see to their training and equipment, and then to ship them across the North Atlantic to France. From the point of view of virus spread, however, the resulting ‘troop movement’ was occurring in the wrong direction. The transportation of the doughboys from North America to France in late 1917 could not have facilitated the passage of a virus ‘from Europe to New York City’.

We set out to resolve this seeming contradiction. In the first place, we reflected once again on the implications of our own hypothesis – the hypothesis that, underlying the ‘minor epidemic’ which occurred at Etaples, was the pathogen which resulted in the 1918 Pandemic. After April 1917, we suggest, it was simply lying low: or, more precisely, maintaining itself ‘in small civilian and military outbreaks while increasing virulence in a stepwise manner’.^77^ But, if such had been the case, the question arises as to how and where these small outbreaks in fact were taking place. And, because we have found no record of such outbreaks in the publications of the day, it is incumbent upon us, as it was indeed on Olson, to suggest a route whereby the virus might possibly have moved from northern France to New York.

At first glance, no answer to this challenge came easily to hand. We looked, of course, at the records of the encampments at Etaples and Rouen. We looked at likely groups of people – soldiers, nurses, and the like – who, having first been thoroughly exposed, might then have served to spread the virus. There seemed an embarrassment of choice. After all, in the spring of 1917, thousands of British, Irish, Australian, Canadian, South African, and other soldiers were passing through these camps, as well as the doctors, the nurses, and other specialists who accompanied these troops. But it seemed impossible for them to have made the journey to Canada or the US East Coast. At both Rouen and Etaples, men and women who fell ill remained bound by their terms of engagement. If they made a rapid recovery, they were returned to duty again; or, if their condition were slow to progress, they were sent to hospitals in England. Travel they might, but only back and forth along the simplest of lines. The same held true for the doctors and the nurses who, more than any others, were exposed to the disease. They, too, were subject to the same rules which restricted every serving infantryman in France. Once attested, and sent across the Channel, they could be moved between the front line and the base; they could be sent to other theatres of the war; they could even go on leave. There seemed, therefore, in the spring of 1917, little opportunity for anyone doing duty at Etaples to thereafter cross the ocean to New York.

We then turned to the records of the United States government itself. Washington had declared war in April 1917, but, given the paucity of soldiers, not a single fighting unit could be sent to France forthwith. A programme of recruitment and training had first to be pushed through. Accordingly, as a US Army staff officer points out, ‘[t]he average American soldier who went to France received six months of training in this country before he sailed’.^78^ That said, these soldiers could not have embarked until the supply lines, the encampments, the dumps, the hospitals, and so forth, had been set up to receive them. Of necessity, therefore, throughout 1917, the American Expeditionary Force sent its experts to France, to listen, to learn, and to put their own installations in place. Indeed, within eight weeks of the United States entering the war, staff officers were heading for Etaples. Major-General H.D.L Parsons, the man responsible for supplying ammunition to every British gun and rifle in action at the Front, takes up the story.

‘Was called to a conference at the Q[uarter]. M[aster]. G[eneral]’s Office to meet three Officers of the “Q” staff of the United States forces who had come over to Europe with General Pershing. I had some conversation with these Officers who were anxious to get all the information we could give them gained by our experience’.^79^

A month later, on 19 July 1917, one such officer called on General Parsons at Etaples.

‘I took Major Drain … to No. 19 Ordnance Depot which we went round in a train, and he saw a sample of each sort of ammunition shelter which we use. He was again considerably impressed …’.^80^

Doubtless, the major was impressed: for here, at No. 19, were stacked more shells and ammunition than the world had ever witnessed in one place.^81^ Drain, however, did not hasten to New York. He returned, instead, to Paris, where he and General Pershing’s other specialists were based.

## The identification of a possible route linking Etaples and New York

Finally, we turned to the army hospitals which had been located at Etaples, the very ones, of course, where Hammond had been conducting his research. Overall, they were under British military control – even the two Canadian general hospitals which were stationed in the camp. The Canadian officers and nurses who were employed were no more free to travel home than were their British counterparts. They had signed ‘for the duration’. On looking closely at their sojourn at Etaples, however, we were struck by a remark which one of their commanding officers had made – less a remark than a critical aside. Whilst walking through the camp on 2 July 1915, shortly after his arrival, Colonel Cameron encountered ‘strangers’ who, like him, had crossed the North Atlantic. These, though, were men and women who, in his view, should not properly be there. More than a hundred doctors and nurses from Chicago had volunteered to do service at Etaples, and they were joined, very soon thereafter, by an equal number from Boston, Massachusetts – the so-called ‘Harvard Unit’.

‘The wisdom of such a step seemed questionable to the Canadians, especially when the national flag of a nation not at war was hoisted at the hospital, where everyone including the strangers was paid by the British Government’.^82^

National rivalries aside, Cameron was pointing to a situation questionable under international law, a situation in which the citizens of a neutral Power were taking part in another country’s war. For unlike their equivalents today, whereby a non-governmental organisation might despatch a hospital to a zone of conflict, to tend the wounded and the sick, these Harvard volunteers were tantamount to serving British personnel. It was not ‘the Harvard Hospital’ they served; it was No. 22 General Hospital, British Expeditionary Force. In one respect, however, they differed from their British and Canadian counterparts. The army’s need for medical assistance, coupled with its wish to encourage the flow of volunteers, had led it to grant the Harvard personnel conditions of employment less onerous than those enforced on everybody else. After six months’ service, these men and women were free to leave. Some of them did leave and had to be replaced. The flow of reinforcements was supervised by the President of Harvard University himself. By 31 May 1917, more than 350 individuals had been identified as having travelled to do service at Etaples.^83^ Some left the hospital, ‘on completion of contract’, and took ship for New York, while others, once returned to North America, chose to volunteer again. A significant number made the journey several times. And, of course, these Harvard men and women were placed at centre stage when purulent bronchitis started causing sickness at the camp. So much so that some of them went down with respiratory disease.

The story of one such surgeon will illustrate this point. Lieutenant Henry Potter, Pennsylvania-trained but working in Rhode Island, reached Etaples on Friday, 8 December 1916. He had travelled with five doctors and twenty nurses, their mission being to replace those members of the Harvard Unit – ten doctors and twice that many nurses – who, their contracts finished, were departing from the base. As the weeks drew on, Henry Potter and his circle succumbed to the illnesses which were circulating at Etaples. In January 1917, he himself fell ill and was hospitalised for upwards of ten days. In February, as he described it to his diary, ‘Sixteen days steadier cold than France has known in the memory of anyone living. 20°F outside. Everybody has a cold. Smith is down with pneumonia’.^84^ Also falling ill were the doctors, nurses, and orderlies who worked in the other hospitals at Rouen and Etaples. These British Army personnel were permanently placed. The men and women of the Harvard Unit, though, were free to move at will: to Scotland, to Bogota, to Greenland, to the south of France – destinations chosen, during the early months of 1917, by Henry Potter’s friends.^85^ To these destinations, they may well have carried pathogens encountered at Etaples.

The story of the Harvard Unit is of particular significance, as the logic of Olson’s hypothesis unfolds. These physicians, surgeons, and nurses, most of them graduates of the Harvard Medical School or connected with the wider medical community in Massachusetts, were on duty at Etaples when, in the early months of 1917, a ‘minor epidemic’ of respiratory disease was sweeping through the camp. Soldiers were dying in the very hospital, No. 22 General, where the Harvard doctors and nurses were providing every element of care, and then these same personnel were saying their goodbyes and sailing westwards to New York. Table 1 provides a list of Harvard volunteers known to have left Etaples during the period from 01 February to 19 March 1917 – the period during which the ‘156 consecutive necropsies’ were being carried out – and to have set off for New York. (We shall never know, of course, whether these individuals were carrying the virus.)^86^ As can be seen, only a few days might elapse between a sojourn in the tented wards at No 22 General Hospital, Etaples, and a cabin on a North Atlantic liner. This would have been within the incubation period and the illness time of newly emerging influenza: a period necessarily more protracted than that described for the fully adapted pandemic strain present in 1918. The respiratory symptoms (except for heliotrope cyanosis) would hardly have been distinguished on a trans-Atlantic voyage from the normal events of winter influenza illness. And neither would the virus have been particularly infectious.

**Table 1.** North American medical and nursing staff who left Etaples during the epidemic of respiratory disease (February–March 1917) and thence travelled to New York Table 1. long description.

Name	Profession	Date of arrival in England	Age on arrival	Sick during the ‘minor epidemic’?	Date of leaving Etaples	Date of leaving England	To US via ship	Departed from	Disembarked at
Payson, Grata	Nurse	31.05.16	23	Yes	09.02.17	17.03.17	Orduna	Liverpool	New York
Robinson, Carl	Surgeon	31.05.16	29	Yes	05.03.17	17.03.17	Orduna	Liverpool	New York
Pike, Forest	Surgeon	30.11.16	42	No	07.03.17	13.03.17	Andania	Liverpool	New York
Provandie, Paul	Surgeon	30.11.16	41	No	07.03.17	13.03.17	Andania	Liverpool	New York
Parker, Harrison	Dentist	31.05.16	27	No	09.03.17	13.03.17	Andania	Liverpool	New York
Dean, Vera	Nurse	31.05.16	27	Yes	09.03.17	17.03.17	Orduna	Liverpool	New York
Golden, Katherine	Nurse	31.05.16	29	No	09.03.17	13.03.17	Andania	Liverpool	New York
Darling, Walter	Surgeon	30.08.16	43	No	09.03.17	17.03.17	Orduna	Liverpool	New York
Sears, Jesse	Nurse	31.05.16	27	No	09.03.17	17.03.17	Orduna	Liverpool	New York

## The role of virus mutation

Today, the capacity of a virus to undergo mutation is better understood. Experimental studies designed to engineer mutations in a virus, studies which are known as ‘gain of function’, have shown that a mere four mutations, out of a total of 12,500, can enable the influenza H5N1 virus to become spreadable from animal to animal.^87^ Of course, given the state of science one hundred years ago, and our inability, to date, to log the mutations inside the H1N1 virus, we can do no more than sketch the outlines of a thesis. The hypothesis, that is, that men and women carrying an early version of the H1N1 virus left Etaples at the height of ‘a minor epidemic’; that they crossed the North Atlantic and landed in New York; and that they then dispersed to their hospitals, their clinics, and their homes. At this point, the virus caused a distinctive pathology only in the bronchi and the lungs; there was little replication in the upper airways. Once contracted, its effects could certainly be lethal, as it spread downwards to the bronchi and lungs, but, on the other hand, it had little capacity to spread. Its survival, once dispersed through North America and confined to isolated pockets, was very much in doubt. It could so easily have disappeared – as seemed to be the case in northern France, once spring got underway.^88^ In mid-1917, though, the United States itself was undergoing a demographic upheaval. In April, that country had entered the war and geared itself up in respect both of industrial production and of the relentless drafting of young men. By the autumn, thirty-two camps and cantonments had been established, and, via a mixture of hutments and tents, ‘shelter’ was provided for practically two million.^89^ The doughboys were young, infection-naïve, and thoroughly confined: ideal conditions in which a virus might proliferate and spread. In this environment, we think, the virus acquired additional mutations: two in the spike protein, and a similar number in other genes. The change was subtle but rendered the virus almost impossible to stop. Scientifically, the virus had exchanged lethality for spreadability. As H1N1 it remained, but, although reduced in its lethality, it could now move easily from one person to the next. Before long, it surfaced in New York, where its impact has been described by Olson. The events which followed have often been related. More than one hundred thousand men sailed for Europe during every month of 1918,^90^ carrying with them the pathogen which had spread throughout their camps. They, and the virus, landed on French shores, and an episode unique in the history of infectious disease was unexpectedly created. There was thus established a complete circle of infection. By the time the main wave was affecting Europe, in October 1918, the case fatality had dropped from over forty per cent to a mere five per cent, at which figure it remained for the following eighteen months.

## The search for preserved lung tissue is long overdue

Here, then, this sketch of our hypothesis must stop. The events which followed as the year 1918 drew on have been logged by diarists, letter-writers, and chroniclers – chroniclers, in fact, whose numbers continue to increase. And yet, regrettably, the raw material which alone could prove or disprove the truth of ours and other theses remains unearthed and undisturbed. That raw material lies in blocks of human tissue: the gold standard, in Olson’s words, for identifying a precursor epidemic wave.^91^ For we remain convinced that, in various repositories, further tissue samples must assuredly survive. In regard to the existence of such samples, we have had direct experience ourselves. We have worked, for instance, in the archives for the Royal London Hospital, where, during the Great War, pathologists took tissue samples from those who perished on its wards. The policy might be indefensible today, but whether right or wrong, a resource was bequeathed to future generations. That resource underwent disruption in 1940, when the hospital was bombed. Even so, some twenty years ago, tissue samples from the Royal London played an important role in identifying the H1N1 virus as the cause of Spanish influenza.^92^

Such repositories are not confined to public institutions. A chance encounter, some fifteen years ago, with a family with long-standing medical connections, brought forth the revelation that, lying in their attic, there were glass slides of human tissue. These had been assembled, during the Great War, from dissections conducted by a physician who did duty at Etaples. The scraps of writing on each label, when lined up with the hospital diaries and the records which Major Guinness had compiled, enabled exact identification of some patients from whom the samples had been taken. To date, technology does not permit the slides to be prized open without the annihilation of their contents, an obstacle which may yet be overcome.^93^

We do not know how many collections of tissue samples were in existence at the end of the Great War. Nor, surprisingly, do we have any information as to the number which still exist today. One can be certain, though, that the second number is but a fraction of the first. There are many reasons why this should be the case. Cities have been bombed, occupied, and suffered changes of regime; and everywhere, old hospitals have been knocked down and new ones erected in their stead. And, to hospital administrators, these collections have sometimes proved a burden. The teaching of pathology today is accorded less priority than, in 1917, was certainly the case, and, equally, the conservation of collections is never likely to be cheap. When found and analysed, tissue samples taken during the years 1916, 1917, and 1918 could provide definitive data about the nature of the virus and enable us to track changes in its pathogenicity and genetic composition.

Finally, a thought on what could have happened, had the world been alerted to the prospect of a possible pandemic, a year or more before it actually arrived. The authors of this paper have referred to the apparent failure, by Drs Bradford and Hammond, working in France, and Drs Abrahams and French, working in the south of England, to give consideration to the notion that, as of early 1917, an influenza pandemic might be imminent. The question still arises, though, as to what might otherwise have happened had Bradford and Abrahams set down their underlying fears in print. Suppose, it could be asked, that the idea of an influenza outbreak had actually been mentioned, what, in the world of 1917, could scientists have done? For, in that year, there was no understanding of what a virus might consist of, and no drugs or intensive care facilities which could potentially save lives. Warnings propagated by a scientific paper would have been redundant and might have fostered panic and alarm. In our view, though, to agree with such a sceptic is to confuse two types of understanding. We accept the fact, of course, that if the word ‘influenza’ had been used, then, since scientists could neither see it nor detect it, the nature of the pathogen could not be understood. But, if one takes a clinical approach, we suggest that an understanding of a different order might quickly have been reached. After all, it was realised from the outset that it was not the ‘influenza’ which was killing people, whatever that entity might be. Rather, it was a range of secondary bacterial invaders which brought about the deaths. Entering the lungs and other organs, once the ‘influenza’ had disarmed the body’s conventional defence, these invaders – the streptococci, the pneumococci, and the rest – were soon present in sufficient numbers and also deeply lodged. They were the cause of the lung blocks, the organ failures, and the fatal terminations. And these invaders were known and understood.

There were, in fact, brave attempts to capitalise on this second type of understanding. Once the pandemic had gained a hold worldwide, experimenters here and there launched efforts to inoculate certain groups of people: units under military discipline, for instance. Such a group could be divided into two: one to be given a set amount of vaccine, the other to be used as a control; and, given the element of discipline involved, the fate of every individual could be tracked. This approach undoubtedly saved lives. The vaccinated group frequently fell ill, but a lesser number went on to fatal termination.^94^ The influenza virus certainly gained entry, but the secondary invaders were halted. These experiments were not initiated until towards the end of 1918, and, in any case, there was no machinery in place to create and to administer the hundreds of millions of doses which a worldwide intervention would have required.

Here we encounter a coincidence, though, which, had it been made use of, would probably have saved lives. In 1917, there was no scientist, anywhere, with greater experience in tackling these secondary invaders than was Sir Almroth Wright. Wright had been engaged by the mine-owners in South Africa, some five years earlier, to investigate the incidence of pneumonia amongst their workers, and to institute a programme of inoculation to stem the havoc which it wrought. Men employed in the mines were contracting pneumonia, and they were dying in some numbers. Wright tackled this head on, albeit using methods which would not be acceptable today. At the outset, in what he termed ‘controlled reconnoitring experiments’, labourers were lined up in a queue. The first man was inoculated with a dose of pneumococcal vaccine, the second given nothing, and so on down the line.^95^ This experiment was greeted with success. In due course, therefore, tens of thousands of mine-workers were treated with these vaccines. At the start, doses of 200 to 300 million units were being used,^96^ but, as time went on, he tried other doses too, up to 2,500 million – despite the fact that ‘inoculations undertaken with doses of over 1000 millions of pneumococci may perhaps temporarily increase the incidence-rate of pneumonia’.^97^ The outcomes, generally, achieved what the mine-owners required: in that ‘the uninoculated had an incidence-rate nearly three times and a death-rate five times greater than the inoculated’.^98^ And thus, whatever reservations one may have about his methods, Wright established inoculation against bacterial pneumonia as a prophylactic.^99^

Wright spent the years of the Great War writing and advising on various infections, from his position as head of a laboratory attached to a British Army hospital in Boulogne – a town on the French coast, not a day’s march from Etaples.^100^ And he had, as his assistant, the microbiologist Alexander Fleming, later to become famous as the discoverer of penicillin. There was nothing Wright’s team could not have produced, had they been asked to prepare vaccines on an industrial scale. Instead, though, they spent the war years investigating the treatment of infections developing in wounds: valuable work, in the context of the time, but a lesser contribution than, in the course of a serious pandemic, they might possibly have made.^101^

## Conclusion

Two papers published in *The Lancet* in 1917 have been analysed at length. We have shown how, in the first such paper, Dr Hammond and his colleagues, working in Etaples, encountered a form of purulent bronchitis whose aetiology seemed to be unknown. They set out to research it and, though not discovering its cause, came to the conclusion that secondary invaders were responsible for the deaths of a number of young men. Their work had, as its corollary, an outcome which they could scarcely have foreseen. The logging which they undertook, taken together with the records which the army so carefully compiled, has enabled us, a century later, to initiate yet further studies into the distinctive illness which these hospitals had seen.

The second paper is noteworthy for quite a different reason. Two of its authors, Drs. Abrahams and French, were amongst England’s most experienced medical practitioners, and, a little later, both men played leading roles during the main wave of the pandemic. Indeed, in October 1918, just as the scourge was coming to its peak, they described what they judged the underlying pathogens to be, and they showed how, in their professional opinion, the condition, which now was killing millions, was closely linked to what they themselves had witnessed, at Aldershot, in the spring of 1917. The ‘purulent bronchitis’ to which both they and Hammond had pointed to in 1917, they wrote, had since been the subject of ‘extended bacteriological research’. In their view, that condition had been ‘primarily influenzal’. Accordingly, it seemed to be akin to what was occurring ‘at the present moment’ (October 1918). After all, they wrote, it was ‘influenzo-pneumococcal septicaemia’ which was ‘responsible for much, if not all, of the fatal “influenzal pneumonia”’ being seen worldwide. Taking the long view, ‘even at the risk of monotonously insistent, we would emphasise our view that in essentials the influenzo-pneumococcal “purulent bronchitis” that we and others described in 1916 and 1917 is fundamentally the same condition as the “influenzo-pneumonia” of this present pandemic’.^102^

So much, then, for what was written at the time. Admittedly, the link between the purulent bronchitis occurring in 1917 and the condition seen during the subsequent pandemic will never be established, unless tissues from the earlier outbreak can be found, tissues in a good enough condition to yield up the genetic evidence required. In this paper, though, we have unearthed evidence of a rather different nature. Taking, as our starting point, the conclusions to which Daniel Olson and his fellow epidemiologists were drawn, we have sought to re-examine the outbreaks, in 1917, of purulent bronchitis. We have then suggested that, if those outbreaks did constitute a herald wave of the subsequent pandemic, then it might be possible to trace the route along which the influenza virus passed. Of course, the service records of British Army personnel were not designed to facilitate research by virologists and epidemiologists today. And the diaries of No. 22 General Hospital, Etaples, were not penned by its commandant in order for historians to list the to and fro of the Harvard volunteers. Even so, we have sought to show, in respect of only two locations, that the logging undertaken at the British Army’s hospitals and camps, taken together with the individual records of the serving personnel, enables papers published in the medical literature of 1917 to be dissected, case by case, one hundred years after the event. And it has enabled us to demonstrate that if indeed, as we suspect, the spillover of the H1N1 influenza virus, from a bird to a human being, took place in northern France in 1916, then a pathway certainly existed whereby that virus could cross the North Atlantic to New York. Once landed at that port, it could spread in a slow but certain fashion through the crowded cities and the burgeoning encampments of a great nation, now girding up for war. It might mutate, it might change the way it spread, but once it had adapted, it was then ready to take up the role of driving the subsequent pandemic.

We conclude with a suggestion as to how a further programme of research might be usefully pursued. Michael Worobey and others have pointed to the unusual pattern of mortality occurring during the main wave of the pandemic. The ‘actual peak in mortality among young adults occurred precisely in those born from 1889 to 1893’.^103^ It should be possible, we think, to apply this very filter to the deaths occurring in the British Army’s hospitals in northern France in the early months of 1917. The deaths amongst the soldiers from war-related wounds occurred, presumably, at ages that were random. The question then arises as to whether deaths from respiratory disease were so weighted as to occur, on an excess pattern, amongst those aged from twenty-four to twenty-eight.

## Supporting information

10.1017/mdh.2025.10053.sm001 Gill and Oxford supplementary material Gill and Oxford supplementary material

## Footnotes

1

C. Langford , ‘Did the 1918–19 Influenza Pandemic Originate in China?’ Population and Development Review, 31 (2005), 473–505 .

2

G.D. Shanks , ‘No Evidence of 1918 Influenza Pandemic Origin in Chinese Laborers/Soldiers in France’, Journal of the Chinese Medical Association, 79 (2016), 46–826542935 10.1016/j.jcma.2015.08.009.

3

A.W. Crosby , America’s Forgotten Pandemic (Cambridge: Cambridge University Press, 1989); J.M. Barry , ‘The Site of Origin of the 1918 Influenza Pandemic and Its Public Health Implications’, Journal of Translational Medicine, 2, 3 (2004). 10.1186/1479-5876-2-3 PMC34038914733617.

4

D. Olson , et al., ‘Epidemiological Evidence of an Early Wave of the 1918 Influenza Pandemic in New York City’, Proceedings of the National Academy of Science,102 (2005), 11059 10.1073/pnas.0408290102PMC118240216046546.

5
*Ibid.*

6
*Ibid.*, 11062.

7 See our *Statement on Methodology* (Supplementary Material).

8

J.A.B. Hammond , W. Rolland , and T.H.G. Shore , ‘Purulent Bronchitis, a Study of Cases Occurring Amongst the British Troops at a Base in France’, The Lancet,190 (1917), 41–4.

9

A. Abrahams et al., ‘Purulent Bronchitis, Its Influenzal and Pneumococcal Bacteriology’, The Lancet,190 (1917), 377–80.

10

M.T. Kinirons and H. Ellis , French’s Index of Differential Diagnosis, an A–Z (London: Routledge, 2016). ‘With industry and ambition he [French] combined a certain sartorial elegance – for, up to his last round, he preserved the custom of appearing in tail coat, top hat and white spats, a gold-headed cane in his hand.’ G.H. Brown , Lives of the Fellows of the Royal College of Physicians of London, 1826–1925 (London: Royal College of Physicians, 1955), iv, 484–5.

11 Abrahams, *op. cit.* (note 9), 377.

12
*Ibid.*

13
*Ibid.*, 378.

14
*Ibid.*, 377–8.

15 Hammond, op. cit. (note 8), 44.

16 Brown, *op. cit.* (note 10), 392.

17 The National Archives, Kew, London (hereinafter TNA), WO 95/4026, Etaples Administrative District, *Staff Diary* (hereinafter *EAD Staff Diary), passim*; K. Cameron, *History of No. 1 Canadian General Hospital* (New Brunswick, Canada: Tribune, 1938), 301.

18 Hammond, *op. cit.* (note 8), 41.

19
*Ibid.*, 44.

20
*Ibid.*, 43.

21
*EAD Staff Diary, op. cit.* (note 17), 27 January 1917.

22 Obituary notice for T.H.G. Shore, *British Medical Journal*, 2 (1961), 1790.

23 Hammond, *op. cit.* (note 8), 41; *EAD Staff Diary, op. cit.* (note 17), *passim.*

24 Hammond, *op. cit.* (note 8), 43.

25
*Ibid.*

26
*Ibid.*, 41.

27
*Ibid.*, 43.

28 It was Richard Pfeiffer, a pupil of Ludwig Koch, who, in 1892, had first proposed *B. influenzae* (now known as *Haemophilus influenzae*) to be the bacterium which causes influenza.

29 Hammond, *op. cit.* (note 8), 44.

30
*Ibid.*

31
*Ibid.*, 41.

32 Abrahams, *op. cit.* (note 9), 377.

33
*Ibid.*

34
*Ibid.*, 382.

35
*Ibid.*

36 Olson, *op. cit.* (note 4), 11062.

37 L.P. Ayres, *The War With Germany, a Statistical Summary* (Washington: Government Printing Office, 1919), 37.

38
*EAD Staff Diary*, *op. cit.* (note 17), 30 September 1917.

39

D. Gill and J. Putkowski , Le Camp britannique d’Etaples (Etaples-sur-Mer: Musée Quentovic, 1997); D. Gill , ‘No Compromise With Truth: Vera Brittain in 1917’, Krieg und Literatur,V (1999), 67–93 ; F. Lemaire , with D. Gill and J. Ramet , ‘Archéoscopie en bleu d’un camp de la Grande Guerre’, Revue du Nord, 101 (2019), 161–211 .

40 Our correspondence with Major Guinness and his son is now held in the Imperial War Museum, London, SE1, as part of the Jesse Short Collection. R.C. Sherriff, who wrote *Journey’s End*, retained to the end of his days the movement order which Guinness had signed: the order, that is, which had sent the playwright up to the Front. ‘He [Guinness] had difficulty signing the order with his left hand, having recently lost his right arm on the Aisne.’ Letter, C. Guinness to D. Gill, 12 August 1998. (Jesse Short collection, Imperial War Museum, London SE1.)

41
*EAD Staff Diary, op. cit.* (note 17), 1 February 1917–19 March 1917. The woman was a nursing sister serving at No. 22 General Hospital, Etaples. She died on 22 February.

42 Hammond, *op. cit.* (note 8), 43.

43 Underdown is the only soldier whose surname begins with a ‘U’, whose death occurred at Etaples during the period covered by our investigation.

44 Hammond, *op. cit.* (note 8), 43.

45 TNA, WO 363, First World War *‘Burnt Documents’* (hereinafter WO 363), Service Record of No. 14075 Private Harry Underdown.

46 Obituary of Private Harry Underdown, *The Kentish Express and Ashford News*, 24 March 1917.

47

C. Hartwell , N. Pevsner , and E. Wilkinson , Nottinghamshire (London: Yale University Press, 2020), 476–7.

48 WO 363, Harry Underdown, *op. cit.* (note 45).

49 WO 363, Harry Underdown, *op. cit.* (note 45); *The Kentish Express and Ashford News*, *op. cit.* (note 46).

50 Information from Pte Underdown’s death certificate.

51 Hammond, *op. cit.* (note 8), 42.

52 WO 363, *op. cit.* (note 45), Service Record of No. 23/455 Private Walter Scott. ‘Notes on examination, post mortem’, compiled by Captain Tull.

53
*Ibid;* ‘Casualty Form – Active Service’.

54
*Ibid*; ‘Notes on F.M. Card’, reproduced on a ‘Medical Case Sheet’ prepared by Captain J.C. Tull.

55
*Ibid.*

56
*Ibid.*

57
*Ibid.*

58
*Ibid.*

59
*Ibid;* ‘Memorandum’, Infantry Records, York, to 3rd Echelon, GHQ, France.

60 Only on one other occasion, during more than twenty years’ work on the service records of British soldiers from the Great War, have the authors seen such a challenge arise.

61

E.S. Thompson , Influenza or Epidemic Catarrhal Fever (London: Percival, 1890).

62
*Ibid.*, xii.

63

R. Pfeiffer and M. Beck , ‘Weiterer Mittheilungen über den Erreger der Influenza’, Deutsche Medizinische Wochenschrift,18, 21 (1892), 465–7.

64

G. Mathers , ‘Etiology of the Epidemic Acute Respiratory Infections Commonly Called Influenza’, Journal of the American Medical Association, LXVIII, 9 (1917), 678–80.

65 Review of A. Bachmeister, *Lehrbuch der Lungenkrankheiten*, Leipzig 1916, in the *Münchener medizinische Wochenschrift*, 6 March 1917.

66 TNA, WO 95/4093, *War Diary*, No. 3 Canadian General Hospital, 27 May 1918.

67

L. Spinney , Pale Rider, the Spanish Flu of 1918 and How It Changed the World (London: Cape, 2017), 162 .

68

M. Imai et al., ‘Experimental Adaptation of an Influenza H5 HA Confers Respiratory Droplet Transmission to a Reassortant H5 HA/H1N1 Virus in Ferrets’, Nature, 486 (2012), 420–822722205 10.1038/nature10831PMC3388103.

69

J.S. Peiris , M.D. de Jong , and Y. Guan , ‘Avian Influenza Virus (H5N1): A Threat to Human Health’, Clinical Microbiological Review,20 (2007), 243–6710.1128/CMR.00037-06PMC186559717428885.

70

W.G. Macpherson et al. (eds), *History of the Great War Based on Official Documents, Medical Services* , Diseases of the War, vol. 1 (London: His Majesty’s Stationery Office, 1923), viii.

71 Hammond*, op. cit.* (note 8). In Germany, the censorship of medical journals also led to the redaction of hospital names and locations.

72 Bradford had a wide remit as a consulting physician to the British Expeditionary Force. He has, however, left no diary, so it is not always possible to chronicle which hospitals he visited and when.

73 We confined our searches to surnames A to H, so as to bring the project down to reasonable dimensions.

74 Hammond, *op. cit.* (note 8), 43.

75 Certain patterns of mortality accompany diseases which are judged to be endemic, but other and quite different patterns will occur during an epidemic or pandemic. In the case of seasonal influenza, for instance, mortality will rise when the winter months set in, only to fall away with the onset of spring. Thus, in northern Europe, during nine winters out of ten, deaths from influenza will rise slowly to a peak, and then fall steadily away. In the case, though, of epidemics propelled by pathogens less habitually seen, the pattern of mortality may prove harder to predict. Different parameters will rule: parameters such as the contagiousness of the pathogen itself, the mobility of the subject population, and the levels of immunity amongst those whom it happens to infect.

76 Olson, *op. cit.* (note 4), 11062.

77

J.S. Oxford et al., ‘World War I May Have Allowed the Emergence of “Spanish” Influenza’, Lancet Infectious Diseases, 2 (2002), 111 11901642 10.1016/s1473-3099(02)00185-8.

78 Ayres, *op. cit.* (note 37), 25.

79 TNA, WO 95/59, Director of Ordnance Services, *War Diary*, 17 June 1917.

80
*Ibid.*, 19 July 1917. The separate stocks of ammunition had to be stored at great distances apart. Forty-five miles of railway track had been laid within the confines of the depot; and the unloading, the loading, and the storage of the ammunition necessitated a fleet of special trains. A.M. Henniker, *History of the Great War, Transportation on the Western Front, 1914–1918* (London: His Majesty’s Stationery Office, 1937), 217.

81 We infer this from the fact that the British Army was using quantities of ammunition comparable to those employed by the Germans and the French, but, having at its disposal a much smaller geographical terrain, had fewer depots in which to hold its stock. Etaples housed its largest depot, and, whilst hostilities persisted, was holding up to fifty-six thousand tons. TNA, WO 95/59, *op. cit.* (note 79), 5 November 1917.

82 Cameron, *op. cit.* (note 17), 204.

83 TNA, WO 95/4084, *War Diary*, No. 22 General Hospital, *passim.*

84 Henry Potter’s diary and correspondence are held by the Imperial War Museum, London SE1.

85
*Ibid.*

86 The table has been constructed from the *War Diary* of No. 22 General Hospital (*op. cit.* [note 83]) and from the lists of passengers sailing from British ports to the United States. These lists are to be found in the BT27 series at The National Archives, Kew. Further volunteers also left Etaples, but did not immediately take ship.

87 Two of these mutations are in the spike protein, and two are in other genes. Imai, *op. cit.* (note 68).

88 ‘Later, when the frost abated’, Hammond wrote, ‘there was a very striking diminution in the number of cases.’ Hammond, *op. cit.* (note 8), 41.

89 Ayres, *op. cit.* (note 37), 28.

90
*Ibid.*, 99.

91 Olson, *op. cit.* (note 4), 11063.

92

A.H. Reid et al., ‘1918 Influenza Pandemic Caused by Highly Conserved Viruses With Two Receptor Binding Variants’, Emerging Infectious Diseases, 9 (2003), 1249–5310.3201/eid0910.020789PMC303308914609459.

93 The significance of these slides is discussed in M. Worobey , J. Cox , and D. Gill , ‘The Origins of the Great Pandemic’, Evolution, Medicine, and Public Health (2019), 18–25. 10.1093/emph/eoz001 30805187 PMC6381288.

94 One may cite, as an example, the programme initiated by a Canadian physician, reported upon in *The Lancet.* 4,842 soldiers, out of 7,600 in the Winnipeg district, were inoculated, some once and some twice. Amongst those inoculated, ‘17 developed pneumonia and 5 died’. Amongst those who had declined to be inoculated, ‘there occurred 41 cases of pneumonia and 17 deaths’. The first inoculations took place on 20 October 1918. F.T. Cadham , ‘The Use of a Vaccine in the Recent Epidemic of Influenza’, The Lancet, 193 (1919), 885–6PMC152354520311273.

95

A.E. Wright et al., ‘Observations on Prophylactic Inoculations Against Pneumococcus Infections, and on the Results Which Have Been Achieved by It’, The Lancet,183 (1914), 87 .

96
*Ibid.*, 7.

97
*Ibid.*, 91.

98
*Ibid.*

99 See also Y-W Chien , K.P. Klugman , and D.M. Morens , ‘Efficacy of Whole-Cell Killed Bacterial Vaccines in Preventing Pneumonia and Death During the 1918 Influenza Pandemic’, Journal of Infectious Diseases, 202, 11 (2010), 1639–16438 21028954 10.1086/657144PMC3107550.

100 We write ‘a day’s march’ advisedly. The base camp at Etaples was deliberately sited a day’s march from Boulogne, so that it could be reached on foot. The railway system was thus spared the burden of conveying the daily drafts of reinforcements.

101 The most recent biography describes the work which Wright (and Fleming) were doing at Boulogne. It provides, however, no more than an overview. A day-to-day account of the work in their laboratories appears not to have survived, which is surprising, perhaps, in that officers commanding such units were obliged to complete a daily diary. Michael Dunnill , The Plato of Praed Street, the Life and Times of Almroth Wright (London: Royal Society of London Press, 2000), 161–200 .

102

A. Abrahams , N. Hallows , and H. French , ‘A Further Investigation into Influenzo-Pneumococcal and Influenzo-Streptococcal Septicaemia: Epidemic Influenzal “Pneumonia” of Highly Fatal Type and Its Relation to “Purulent Bronchitis”’, The Lancet, 193 (1919), 1 .

103

M. Worobey , G-Z. Han , and A. Rambaut , ‘Genesis and Pathogenesis of the 1918 Pandemic H1N1 Influenza A Virus’, Proceedings of the National Academy of Science, 508 (2014), 8110 10.1073/pnas.1324197111PMC405060724778238.

## Supplementary material

The supplementary material for this article can be found at http://doi.org/10.1017/mdh.2025.10053.

## Long descriptions

### Chart 1. Long description

The y-axis is labeled Number of deaths per week and ranges from 0 to 7. The x-axis is labeled Calendar weeks, 1 October 1916 to 31 May 1917, with tick marks from 1 to 35.

Data trends:

* Weeks 1 through 9: Zero deaths recorded.

* Week 10: A sudden spike to 4 deaths.

* Weeks 11 through 14: Consistent at 1 death per week.

* Week 15: Increases to 3 deaths.

* Weeks 16 and 17: Return to 1 death per week.

* Weeks 18 to 21: A major cluster of high mortality. Week 18 has 5 deaths, week 19 has 5 deaths, week 20 reaches the peak of 6 deaths, and week 21 has 4 deaths.

* Weeks 22 and 23: Decrease to 2 deaths per week.

* Week 24: 1 death.

* Week 25: 2 deaths.

* Week 26: 1 death.

* Week 27: 3 deaths.

* Week 28: 0 deaths.

* Week 29: 2 deaths.

* Week 30: 0 deaths.

* Week 31: 2 deaths.

* Week 32: 1 death.

* Week 33: 0 deaths.

* Week 34: 1 death.

* Week 35: 0 deaths.

Navigate back to Chart 1..

### Chart 2. Long description

The Y axis is labeled No. of deaths per week and ranges from 0 to 7. The X axis is labeled Calendar weeks, 1 Oct 1916 to 31 May 1917, with numbered ticks from 1 to 36.

Data points by week:

* Weeks 1 through 3: 0 deaths.

* Week 4: 1 death.

* Weeks 5 and 6: 0 deaths.

* Weeks 7 and 8: 2 deaths each.

* Week 9: 0 deaths.

* Week 10: 4 deaths.

* Week 11: 1 death.

* Week 12: 3 deaths.

* Week 13: 1 death.

* Week 14: 3 deaths.

* Week 15: 6 deaths.

* Week 16: 1 death.

* Week 17: 2 deaths.

* Week 18: 0 deaths.

* Week 19: 2 deaths.

* Week 20: 3 deaths.

* Week 21: 6 deaths.

* Week 22: 3 deaths.

* Week 23: 5 deaths.

* Weeks 24 and 25: 3 deaths each.

* Week 26: 2 deaths.

* Week 27: 3 deaths.

* Week 28: 2 deaths.

* Week 29: 1 death.

* Week 30: 2 deaths.

* Week 31: 0 deaths.

* Week 32: 2 deaths.

* Weeks 33 through 36: 0 deaths.

The chart shows two primary peaks at 6 deaths occurring during week 15 and week 21.

Navigate back to Chart 2..

### Table 1. Long description

The table contains ten columns: Name, Profession, Date of arrival in England, Age on arrival, Sick during the minor epidemic?, Date of leaving Etaples, Date of leaving England, To U S via ship, Departed from, and Disembarked at.

* Payson, Grata: Nurse, arrived 31.05.16, age 23, sick Yes, left Etaples 09.02.17, left England 17.03.17 via Orduna from Liverpool to New York.

* Robinson, Carl: Surgeon, arrived 31.05.16, age 29, sick Yes, left Etaples 05.03.17, left England 17.03.17 via Orduna from Liverpool to New York.

* Pike, Forest: Surgeon, arrived 30.11.16, age 42, sick No, left Etaples 07.03.17, left England 13.03.17 via Andania from Liverpool to New York.

* Provandie, Paul: Surgeon, arrived 30.11.16, age 41, sick No, left Etaples 07.03.17, left England 13.03.17 via Andania from Liverpool to New York.

* Parker, Harrison: Dentist, arrived 31.05.16, age 27, sick No, left Etaples 09.03.17, left England 13.03.17 via Andania from Liverpool to New York.

* Dean, Vera: Nurse, arrived 31.05.16, age 27, sick Yes, left Etaples 09.03.17, left England 17.03.17 via Orduna from Liverpool to New York.

* Golden, Katherine: Nurse, arrived 31.05.16, age 29, sick No, left Etaples 09.03.17, left England 13.03.17 via Andania from Liverpool to New York.

* Darling, Walter: Surgeon, arrived 30.08.16, age 43, sick No, left Etaples 09.03.17, left England 17.03.17 via Orduna from Liverpool to New York.

* Sears, Jesse: Nurse, arrived 31.05.16, age 27, sick No, left Etaples 09.03.17, left England 17.03.17 via Orduna from Liverpool to New York.

Navigate back to Table 1..

