# Supplementary material for: The ‘Spanish’ influenza pandemic: new evidence for influenza outbreaks in England and France prior to 1918
Source: Med Hist. 2026 Jul;70(3):429–48. doi: 10.1017/mdh.2025.10053 (PMC13311354; doi:10.1017/mdh.2025.10053)
Supplement: Gill and Oxford supplementary material [file S0025727325100537sup001.docx]

Statement on Methodology

For the period August 1914 to March 1918, we inspected every page of the following journals:

- *Bulletin de l’Institut Pasteur*
- *Bulletin de l’Académie de Médécine*
- *Münchener Medizinische Wochenschrift* (and its supplement, the *Feldärztliche Beilage*)
- *Journal of the American Medical Association*
- *The Lancet*
- *The British Medical Journal*

We were not searching for the key word ‘influenza’; but, rather, for unexplained (and abnormally fatal) outbreaks of respiratory disease. It was through these means, we surmised, that a hitherto unrecognised ‘herald wave’ of the Spanish Influenza Pandemic of 1918-1919 might come to be detected.

Many intriguing papers led to a dead end. There was extensive coverage of influenza *per se* in the pages of the *Journal of the American Medical Association*: this we judged, ultimately, to belong to the class of seasonal influenza. Again, numerous papers dealing with a widespread, non-fatal condition were published in the *Münchener Medizinische Wochenschrift*: which condition was later judged by contemporary opinion not to be respiratory at all.^[[1]](#footnote-1)^ Two papers alone seemed to fit our notion of how a herald wave might present itself, before the main wave had arrived. Both were published by *The Lancet*, and both concerned ‘almost a minor epidemic’ of purulent bronchitis in the year 1917.

In regard to the first such paper, emanating from the base camp at Etaples, identification of those cases which had been subjected to examination *post mortem* proved possible, once the unconventional nature of the record-keeping had been grasped and understood. At Etaples, for instance, deaths through disease were meticulously logged, and, over and above this logging, the diaries of each British army hospital (in all, providing more than twenty thousand beds) have been carefully preserved. Armed with the names of those who died, we then searched the records of service for these ‘officers and men’: which records, when preserved, furnish salient details about sickness and demise. Beyond that, the usual genealogical tools remained at our disposal. Perhaps the most important lay in the archives of the Commonwealth War Graves Commission. The Commission stands as the guardian of those who came to be interred; which interment inevitably took place in the cemetery closest to the hospital concerned.

The Harvard personnel worked exclusively at No. 22 General Hospital, Etaples. The diary of that hospital’s commandant provides a daily log of the arrival and departure of this personnel: it seems to be complete, though it is possible that some doctors and nurses may have come and gone without being properly recorded. The passenger lists, both ‘Inwards’ and ‘Outwards’, in regards to trans-Atlantic crossings, are to be found in The National Archives, Kew; and though the ‘inwards’ records provide an exact tally of those destined for No. 22, the ‘outwards’ records can offer no such certainty. After all, numbers of the Harvard personnel elected for a delayed or circuitous return.^[[2]](#footnote-2)^ The comings and goings of the Harvard personnel were reported on extensively in magazines and local newspapers on the American East Coast; beyond which, many short biographies exist – principally in regard to the surgeons and physicians – in the *American Medical Directory* and the records of Harvard University itself.

Finally, a word of acknowledgement to two colleagues who, over the years, have furnished quantities of help. Whilst working on a wider project, relating to the respiratory ailments in army hospitals in France, the authors engaged Judith Hibbert in the work of researching the medical and service records of the many men who died; she has helped create a database, in addition, of the Harvard personnel. That project has not yet reached fruition. Assistance of a quite different kind was rendered by a colleague in Etaples. The archaeologist Joël Ramet has, for thirty years, been working and dwelling in that town. His familiarity with the landscape across which the British base was built, and his links with local people, have given him an unrivalled knowledge of the sites on which these hospitals once stood, of their supply lines, staff accommodation – of the pits, even, into which their discarded material was thrown. His enthusiasm has spurred our work.

Extracts from Crown copyright material in The National Archives have been used under the terms of the Open Government Licence v3.0.

1. A discussion of these outbreaks will be found in M. Worobey, J. Cox, D. Gill, ‘The Origins of the Great Pandemic’, *Evolution, Medicine, and Public Health,* 2019, 18-25. [↑](#footnote-ref-1)
2. The series BT26 contain the inwards passenger lists; the outward are in BT 27. [↑](#footnote-ref-2)
